# Overweight Management through Mild Caloric Restriction in Multigenerational Long-Tailed Macaque Breeding Groups

**DOI:** 10.3390/vetsci9060262

**Published:** 2022-05-31

**Authors:** Dian G. M. Zijlmans, Annemiek Maaskant, Annet L. Louwerse, Elisabeth H. M. Sterck, Jan A. M. Langermans

**Affiliations:** 1 Animal Science Department, Biomedical Primate Research Centre, 2288 GJ Rijswijk, The Netherlands; maaskant@bprc.nl (A.M.); louwerse@bprc.nl (A.L.L.); e.h.m.sterck@uu.nl (E.H.M.S.); langermans@bprc.nl (J.A.M.L.); 2 Animal Behaviour and Cognition, Department of Biology, Utrecht University, 3508 TB Utrecht, The Netherlands; 3 Department Population Health Sciences, Unit Animals in Science and Society, Faculty of Veterinary Medicine, Utrecht University, 3584 CM Utrecht, The Netherlands

**Keywords:** dietary restriction, nutrition, welfare, obesity, reproduction, group-living

## Abstract

Caloric restriction (CR) is an effective method to reduce overweight in captive non-human primates (NHPs). CR has been applied to individually- and pair-housed NHPs, but whether applying CR can be effective and safe in group-housed NHPs has not yet been assessed. This study investigates the effect of mild (20%) CR on adult overweight and biochemical parameters, immature growth, veterinary consultations, and reproductive success in multigenerational long-tailed macaque (*Macaca fascicularis*) breeding groups. Data were derived from anthropometric measurements and blood samples during yearly health checks, complemented with retrospective data on veterinary consultations and reproductive success. Adult body measures decreased after CR, with heavier individuals and females losing more weight compared to leaner individuals and males. CR lowered cholesterol levels in adults but had no overall effect on other biochemical parameters. Yet, biochemical parameters of individuals with high baseline values were reduced more compared to individuals with low baseline values. Immature growth, veterinary consultations and reproductive success were not influenced by CR. Thus, CR targeted the right individuals, i.e., overweight adults, and had no adverse effects on the variables examined in this study. This implies that mild CR can be a valuable overweight management strategy in group-housed NHPs.

## 1. Introduction

Non-human primates (NHPs) are susceptible to developing overweight in captivity, even when maintained on a nutritionally balanced diet [1]. Many primate facilities feed their social groups ad libitum or provide a predetermined amount of food that is available throughout the day [2]. This way of feeding has likely contributed to the increasing overweight prevalence among group-housed NHPs. In research facilities, overweight prevalence of group-housed NHPs roughly varies between 10 and 30% of the individuals [3,4,5,6,7]. This overweight prevalence may continue to rise, as little research and information are available on overweight prevention and treatment in group-housed NHPs [8]. Primate facilities should remedy overweight, as overweight can cause similar health problems in NHPs as in humans, e.g., type 2 diabetes mellitus and cardiovascular disease [9,10,11].

An effective method to prevent overweight and overweight-related health problems in NHPs is caloric restriction (CR), i.e., a nutritional intervention that reduces caloric intake without malnutrition [9,12]. Beneficial effects of CR on health- and lifespan have been found in several NHP species, among others in long-tailed macaques (*Macaca fascicularis*: [13]), rhesus macaques (*Macaca mulatta*: [9,14]), squirrel monkeys (*Saimiri sciureus:* [15]) and grey mouse lemurs (*Microcebus murinus*: [16]). The animals in these studies were housed individually or in pairs due to the experimental need to control and quantify food intake. One study performed CR in group-housed NHPs and found that baboon mothers (*Papio* spp.) subjected to CR during pregnancy weighed less than ad libitum fed control mothers [17]. However, these baboons were trained to feed in individual cages [18], impeding social interactions around food intake. Although CR seems to be a promising method to prevent and decrease overweight without separating individuals from their social group, its use has not yet been validated for NHPs housed and fed in social groups.

Some researchers argue that CR may not be appropriate or feasible in social groups [8], because food is often not equally divided among group members (e.g., [19,20]). This is especially important in multigenerational breeding groups, because immatures need enough energy to enable growth. CR reduces the number of calories and therefore has the potential side-effect to restrict immature growth. Growth rates of immature yellow baboons (*Papio cynocephalus*) are lower in completely wild-feeding than in partially food-enhanced (i.e., 4.9 g/day vs. 8.7 g/day) social groups [21]. Offspring growth was also slower in grey mouse lemur infants of calorie restricted mothers compared to infants reared by ad libitum fed mothers [22]. Thus, CR may slow immature growth.

CR may also negatively affect other, especially low-ranking, individuals, as CR likely increases feeding competition. High-ranking individuals typically have priority of access to preferred resources such as food [20,23,24]. They can fulfil their energetic requirements during CR by monopolizing food, while low-ranking individuals cannot. In wild Japanese macaques (*Macaca fuscata*), metabolizable energy intake was lower in low-ranking females compared to high-ranking females in times of food scarcity, resulting in energy shortage and three lower-ranking females dying [25]. Besides, several studies have shown that aggressive behaviour of NHPs increases when the availability and distribution of food are restricted [23,26]. CR may therefore result in more veterinary consultations as competition can potentially lead to severe weight loss and increased number of injuries or even mortality.

Besides maintaining stable groups and maximizing the animals’ health and welfare, another important aspect of managing NHP breeding colonies is to produce offspring. Reproduction is energetically costly and reproductive success is sensitive to a limited food supply [27]. After periods of drought and extreme heat, when food becomes scarce, wild female yellow baboons were less likely to cycle, conceive and give birth to living infants [28]. Similarly, in wild Japanese macaques, only one (high-ranking) female produced offspring after a food-scarce season, while twelve females did so when there was no food scarcity [25]. In wild long-tailed macaques, increased food supply during highly productive mast years resulted in higher birth rates and earlier conception [29]. In captive studies, CR inhibited ovulation in rhesus macaques [30], and even induced pregnancy loss in common marmosets (*Callithrix jacchus*: [31]). Thus, CR may lead to reduced reproductive success, which would be an undesirable outcome.

To the authors’ knowledge, it is currently unknown whether applying CR can be effective and safe in group-housed NHPs. CR should ideally target overweight adults, while having no adverse effects on other group members. We investigate the effect of mild (20%) CR on adult overweight and biochemical parameters, immature growth, veterinary consultations, and reproductive success in multigenerational long-tailed macaque breeding groups, thereby aiming to determine the suitability of CR as an overweight management strategy in group-housed NHPs. Anthropometric measurements and blood samples were collected during annual health checks. Adult overweight and immature growth were derived from body weight and height measurements. Blood samples were analyzed for several biochemical parameters related to lipid metabolism and glycemic response. Veterinary consultations and reproductive success were derived in retrospect from the year-round electronic health records.

## 2. Materials and Methods

### 2.1. Animals and Housing

Ten multigenerational long-tailed macaque (*Macaca fascicularis*) breeding groups from the Biomedical Primate Research Centre (BPRC) in Rijswijk, the Netherlands, were examined for this study. The breeding colony housed approximately 200 animals (10–40 animals per group) throughout the study period, ranging between 0 and 24 years of age. Natural group dynamics were adhered to when the groups were formed, i.e., females are philopatric and males leave the natal group at puberty. The multigenerational groups consisted of adult females from several matrilines with their (mature) offspring and one (or two) unrelated breeding male(s). Breeding males were almost continuously present, except for some short periods between the removal of an old breeding male and the introduction of a new one. Not every individual in the breeding colony was included in all statistical analyses due to varying reasons (see Section 2.4).

The breeding groups were housed in enriched enclosures with free access to both indoor (±72 m^2^ and 2.85 m high) and outdoor (±250 m^2^ and 3.1 m high) compartments. The indoor compartments contained sawdust, while the outdoor compartments had a sand floor where natural plant growth was possible. The enclosures contained several climbing structures, fire hoses, car tires, beams, sitting platforms and an outside swimming pool [32]. Water was available throughout the day from automated water dispensers. Experienced ethologists and colony managers regularly observed dominance interactions in the groups and assigned rank categories to all females based on their knowledge and experience with the groups. Females were categorized as being high-ranking (H), middle-ranking (M) or low-ranking (L) within their social group. Roughly one-third of each group was assigned to each category, while taking into consideration the matrilineal structures. Adult breeding males were always considered high-ranking.

### 2.2. Diet and Caloric Restriction

The daily diet of the macaques started with monkey chow (Primates/NHP–pellets, Ssniff, Soest, Germany) in the morning. The amount of monkey chow was calculated using the basal metabolic rate (BMR), which depended on the age, sex and body weight of each individual [33]. The monkey chow provided all the daily required energy per individual and was provided in multiple feeding trays per group (i.e., typically six trays per group, but at least one tray per five animals with a minimum of two trays) to prevent monopolization. In addition, fresh fruit, vegetables, bread or a grain mixture were presented in the afternoon. For fruit and vegetables, 150 g was prepared for each individual aged four years and older, and 100 g for individuals between one and three years old. One slice of bread and 20 g of the grain mixture were counted per animal. Food enrichment and treats were provided occasionally. The daily diet was accessible for all group members. In this situation, overfeeding likely occurred as the monkey chow provided all required energy per individual and other items were provided in addition.

Due to the relatively high prevalence of overweight in the breeding colony (i.e., 22%), the veterinary team decided to initiate mild CR in August 2017, reducing the provided number of calories by 20% (Table 1). The amount of monkey chow based on the BMR was multiplied by 0.8 to obtain the new amount per individual. Since the feeding trays regularly contained left-over pellets the following morning before CR [34], we consider 20% to be a reasonable reduction, while still providing enough food to meet energetic requirements and prevent malnutrition. Additionally, the amount of fruit and vegetables was reduced from 100/150 g to 80/120 g per individual and the grain mixture from 20 g to 15 g per individual. The number of bread slices remained equal after CR.

### 2.3. Data Collection

#### 2.3.1. Health Checks

Anthropometric measurements and blood samples were collected once per year during annual health checks. These health checks are a routine veterinary procedure related to the regular health management of the colony (cf. [35]). Individuals were fasted overnight and sedated with an intramuscular injection of ketamine (10 mg/kg, Ketamine 10%; Alfasan, Woerden, The Netherlands) prior to the health check. Individuals younger than eight months old were not sedated and were left with their mother during the entire procedure (if possible). The health checks before CR took place in November 2016 or spring 2017, while the health checks after CR took place in spring 2018.

Anthropometric measurements of full-grown adults and immatures were collected as previously described [5]. Body weight was measured with a standard scale. Height was measured as crown-rump length with the animal in a supine position using a measuring mat for human infants (SECA, Hamburg, Germany). Abdominal circumference was measured at the level of the umbilicus with the animal in a supine position using a flexible tape measure. Body weight was measured for all individuals, whereas height and abdominal circumference could not always be accurately measured, e.g., when individuals woke up from their sedation. Body weight, height and abdominal circumference were measured to one decimal point. Overweight status of adults was based on a species-specific weight-for-height index, known as WHI2.7 [5], hereafter referred to as WHI. This was calculated as weight in kilograms divided by height in meters to the power of 2.7. WHI of group-housed long-tailed macaques ideally ranges between 39 and 62 kg/m^2.7^ [5]. Individuals with a WHI below the lower boundary were considered underweight, while exceeding the upper WHI boundary resulted in overweight.

Blood samples of full-grown adults were collected from the vena femoralis and analyzed for total cholesterol (mmol/L), triglyceride (mmol/L), glucose (mmol/L), fructosamine (umol/L) and glycated hemoglobin (HbA1c; %) levels using a Cobas Integra 400 plus (Roche Diagnostics, Rotkreuz, Switzerland). One female had no blood sample available after CR, so she was not included in the analyses on biochemical parameters. Due to technical issues, the HbA1c values determined in spring 2017 were not comparable to the other values, reducing the sample size for this analysis.

#### 2.3.2. Health and Reproductive Records

Animal caretakers monitored the animals twice daily (during the feeding rounds) and reported births and abnormalities in a digital database, which contained the health records of all animals that live at the BPRC. Cases of severe weight loss that required veterinary consultation were obtained in retrospect from the database. The proportion of individuals with severe weight loss before and after CR was calculated as the number of individuals with severe weight loss per year divided by the total size of the breeding colony. Furthermore, we calculated the proportion of individuals with injuries that required veterinary consultation, one year before and after CR. Injuries were included when the integumentary system of the animal was affected, e.g., lacerations, and a veterinarian consult was required. The necessity of a consultation is determined according to a standardized protocol. Finally, we examined cases of mortality when the integumentary system of the animal was affected after a suspected or observed conflict. However, the number of mortality cases was too limited for statistical analyses.

Reproductive success was measured using the proportion of pregnant females and the proportion of successful pregnancies in the year before and after CR. The number of females that conceived was calculated backwards from the number of births based on an average pregnancy length of 164 days [36]. The proportion of pregnant females per year was calculated as the number of females that conceived divided by the total number of fertile females, i.e., females that were sexually mature (>3 years old) and not on contraceptives. Pregnancy outcome was considered successful when the offspring was born alive. The proportion of successful pregnancies before and after CR were calculated by dividing the number of infants born alive by the total number of pregnancies per year.

### 2.4. Data Analyses

The effectiveness of CR was tested by comparing anthropometric measurements and biochemical parameters of full-grown adults (females > 6 years old, males > 8 years old; [37]) before and after CR. To exclude possible confounding factors, adults were used as their own control in a paired design. Some adult males were added or removed from the breeding colony during the study period to prevent inbreeding, so there was limited paired data for adult males. Females that were pregnant during one or both health checks (*n* = 19) were excluded as pregnancy is known to affect body fat levels [37]. Six (pre-)diabetic individuals were excluded to prevent possible confounding effects of disease progression on our outcome measures. Eventually, 41 adult females and five adult males aged between 6 and 22 years old were included in the pairwise comparisons.

The effectiveness of CR on body measures and biochemical parameters of full-grown adults was tested with a paired samples *t*-test or Wilcoxon signed ranks test. A Shapiro–Wilk test was used to test whether the data were normally distributed. A linear regression analysis checked which adult individuals were affected most by CR. The effect of CR, i.e., delta of a measure, was calculated by subtracting the value before CR (in 2016/2017) from the value after CR (in 2018). We tested whether an individual’s age, sex and baseline value were related to delta WHI, cholesterol, triglyceride, glucose, fructosamine and HbA1c. Breeding group was included as a random factor in all models, and normality and homoscedasticity of the residuals were visually checked. Whether female dominance influenced delta WHI and overweight prevalence before and after CR was tested with a one-way ANOVA and Fisher’s exact tests, respectively.

In addition, we checked whether CR had adverse effects on immature growth, veterinary consultations, and reproductive success. Immature growth was derived from cross-sectional body weight and height data of 111 immatures aged between eight months and four years old. Some immatures contributed to the dataset twice (*n* = 58), while others were included only once (*n* = 53), depending on their age. Body weight of three-year old females was excluded when pregnant (*n* = 6). Linear regression analyses checked whether age, sex and CR affected immature body weight and height. Interaction effects were included in the model when they had a significant impact on the outcome measure, and breeding group was included as a random factor.

Finally, data from the health and reproductive records were examined. Whether the number of individuals with severe weight loss relative to the total breeding colony size (*n* = 196 before CR, *n* = 211 after CR) differed before or after CR was tested with Fisher’s exact test. A chi-square test was used to test the proportion of individuals with injuries that required veterinary consultation before and after CR. The effect of CR on female reproduction was tested by comparing the proportion of pregnant females and the proportion of successful pregnancies before and after CR. Only fertile females were included in these analyses (*n* = 74 before CR, *n* = 87 after CR). Chi-square tests and a Fisher’s exact test were used to examine the effects of CR and female dominance on likelihood of conception and pregnancy outcome.

Data analyses were performed in SPSS version 28 and R studio version 1.2.5. All tests were two-tailed and a value of *p* < 0.05 was considered significant, but trends (0.05 ≤ *p* < 0.10) are also reported. Descriptive statistics are presented in the results as mean ± SE.

### 2.5. Animal Ethics

Ethical approval for this study was waived by BPRC’s Animal Welfare Body (Instantie voor Dierenwelzijn). The research involved less than minimal discomfort to the animals used in this manuscript according to the definition of an animal experiment as defined by the European Directive 2010/63/EU. All procedures performed in this study complied with the applicable international, national and institutional guidelines and regulations.

## 3. Results

### 3.1. Adult Overweight

**Figure 1:**
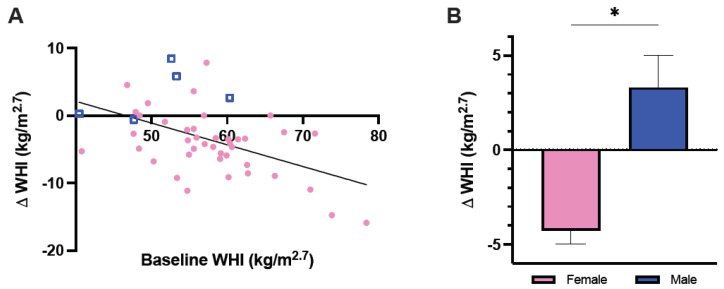
Effect of baseline weight-for-height index (WHI) (**A**) and sex (**B**) on delta WHI in full-grown adult male and female long-tailed macaques. Note that the y-axes are not equally scaled. Circles represent females; squares represent males. Error bars represent the standard error. * *p* < 0.05.

Body weight of full-grown adults significantly decreased from 6.7 ± 0.2 kg before CR to 6.3 ± 0.2 kg after CR (Wilcoxon signed ranks test, *Z* = −3.533, *n* = 46, *p* < 0.0005). Similarly, abdominal circumference (paired samples *t*-test, *t* = 5.960, *n* = 38, *p* < 0.0005) and WHI (paired samples *t*-test, *t* = 4.498, *n* = 46, *p* < 0.0005) decreased after CR (Table 2). Delta WHI was independent of age (F(1,33) = 2.304, *p* = 0.139), but significantly related to baseline WHI (F(1,33) = 6.488, *p* = 0.016) and sex (F(1,33) = 5.201, *p* = 0.029). WHI decreased more in individuals with a higher baseline WHI (Figure 1A). Furthermore, WHI significantly decreased in females, but not in males (Figure 1B). Delta WHI did not differ between females from different rank categories (one-way ANOVA, F(2,38) = 1.927, *p* = 0.160).

Overall, ten individuals (22%; rank category 4H, 3M, 3L) met the overweight criterium before CR, and six individuals (13%; 2H, 4M, 0L) did so after CR. Female dominance did not affect overweight prevalence before CR (Fisher’s exact test, *p* = 0.587) or after CR (Fisher’s exact test, *p* = 0.174). When data were split by sex, overweight prevalence in females went from ten individuals (24%) before CR to five individuals (12%) after CR. In contrast, no adult male was overweight before CR, while one male developed overweight after CR. Underweight was absent from the breeding colony before CR, whereas one high-ranking female (2%) met the underweight criterion after CR.

### 3.2. Biochemical Parameters of Adults

**Figure 2:**
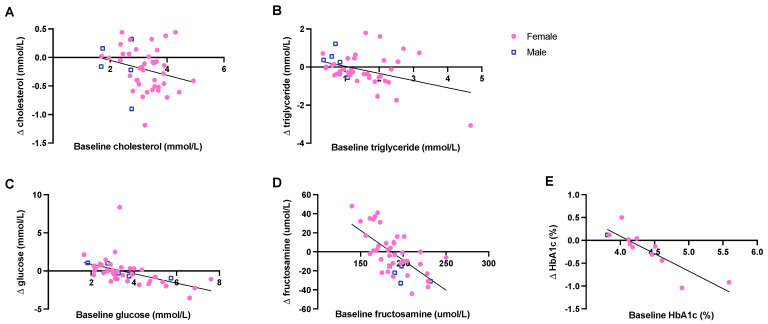
Effect of baseline value on delta cholesterol (**A**), delta triglyceride (**B**), delta glucose (**C**), delta fructosamine (**D**), and delta HbA1c (**E**) in full-grown adult male and female long-tailed macaques. Circles represent females; squares represent males.

Cholesterol levels significantly decreased after CR (paired samples *t*-test, *t* = 3.650, *n* = 45, *p* = 0.001; Table 2). Although triglyceride levels went from 1.46 ± 0.13 mmol/L before CR to 1.33 ± 0.14 mmol/L after CR, this change was not statistically significant (Wilcoxon signed ranks test, *Z* = −1.236, *n* = 45, *p* = 0.216; Table 2). When triglyceride data were split by sex, there was a trend for a significant reduction in triglyceride levels in females (Wilcoxon signed ranks test, *Z* = −1.754, *n* = 40, *p* = 0.079), but not in males (paired sample *t*-test, *t* = −1.294, *n* = 5, *p* = 0.265). Delta cholesterol and delta triglyceride were independent of age (F(1,32) = 0.059, *p* = 0.809; F(1,32) = 1.048, *p* = 0.314) and sex (F(1,32) = 0.401, *p* = 0.531; F(1,32) = 0.654, *p* = 0.425), but there was a trend for a negative association between the delta and baseline values (F(1,32) = 4.125, *p* = 0.051; F(1,32) = 3.377, *p* = 0.075; Figure 2A,B). Thus, individuals with higher baseline values tended to have a higher decrease in cholesterol and triglyceride after CR.

CR had no significant influence on glucose (Wilcoxon signed ranks test, *Z* = −1.230, *n* = 45, *p* = 0.219), fructosamine (paired samples *t*-test, *t* = 0.684, *n* = 45, *p* = 0.498) and HbA1c levels (paired samples *t*-test, *t* = 1.494, *n* = 12, *p* = 0.163; Table 2). Delta glucose was independent of age (F(1,32) = 0.045, *p* = 0.833) and sex (F(1,32) = 0.132, *p* = 0.719), while individuals with higher baseline glucose levels had a higher glucose reduction after CR (F(1,32) = 15.115, *p* < 0.0005; Figure 2C). Similarly, delta fructosamine and delta HbA1c were inversely related to the baseline values (F(1,32) = 15.856, *p* < 0.0005; F(1,7) = 11.930, *p* < 0.0005; Figure 2D,E), but not related to age (F(1,32) = 0.390, *p* = 0.536; F(1,7) = 0.007, *p* = 0.937). Delta HbA1c was also independent of sex (F(1,7) = 0.422, *p* = 0.537), while males had a higher delta fructosamine compared to females (F(1,32) = 8.452, *p* = 0.007). When data were split by sex, fructosamine levels decreased after CR in males (paired samples *t*-test, *t* = 6.063, *n* = 5, *p* = 0.004), but did not differ in females (paired samples *t*-test, *t* = −0.100, *n* = 40, *p* = 0.921). Thus, individuals with higher baseline values had a higher decrease in glucose, fructosamine and HbA1c after CR.

### 3.3. Immature Growth

**Figure 3:**
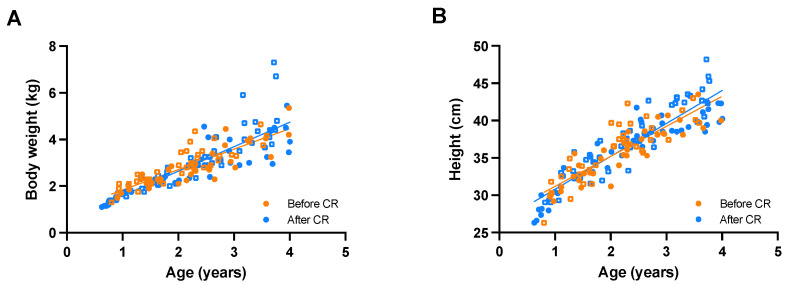
Growth patterns of immature male and female long-tailed macaques based on body weight (**A**) and height (**B**) before and after caloric restriction (CR). Circles represent females; squares represent males.

**Figure A1:**
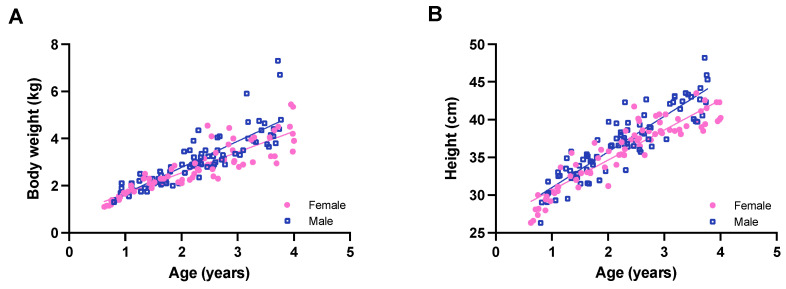
Growth patterns of immature male and female long-tailed macaques based on body weight (**A**) and height (**B**). The steeper blue line implies that the growth rate of immature males is higher compared to females.

Body weight of immatures was independent of sex (F(1,150) = 0.915, *p* = 0.340) and CR (F(1,150) = 1.463, *p* = 0.228; Figure 3A), while body weight was positively associated with age (F(1,150) = 656.152, *p* < 0.0005). Body weight increased on average with 0.99 kg per year. There was also a significant interaction effect between age and sex in the model (F(1,150), 6.740, *p* = 0.010). This means that the relationship between age and body weight differed per sex. The regression equations showed that male body weight = 0.55 + 1.11 × age, while female body weight = 0.78 + 0.88 × age (Figure A1).

Similarly, height of immatures was independent of sex (F(1,148) = 0.418, *p* = 0.519) and CR (F(1,148) = 0.0270, *p* = 0.604; Figure 3B), but increased with age (F(1,148) = 768.054, *p* < 0.0005). On average, height increased with 4.2 cm per year. There was again a significant interaction effect between age and sex (F(1,148) = 4.133, *p* = 0.044). The regression equations showed that male height = 26.27 + 4.73 × age, while female height = 27.1 + 3.85 × age (Figure A1). Thus, the age–sex interaction effects show that male immatures have a higher increase in body weight and height per year compared to female immatures, meaning males grow faster than females, while CR had no significant effect on immature growth.

### 3.4. Health Records

Severe weight loss that required veterinary consultation occurred in three individuals (1.5%) before CR and seven individuals (3.3%) after CR. The proportion of individuals with severe weight loss did not differ significantly before or after CR (Fisher’s exact test, *p* = 0.341). Besides, five of these individuals had a confirmed diagnosis of diabetes mellitus, while five other cases of severe weight loss were linked to chronic or intermittent diarrhea (Table A1). Thus, the severe weight loss of these individuals was likely caused by underlying medical conditions, rather than by CR.

Six individuals (3.1%) were seen by the veterinarian for injuries prior to CR and six individuals (2.8%) after CR. The nature and location of most injuries were suggestive to be inflicted by other group members. The proportion of individuals with injuries that required veterinary consultation did not differ after CR (chi-square test, χ^2^ = 0.017, *p* = 0.897). In addition, mortality due to suspected or observed conflicts occurred twice in the year before CR and once in the year after CR. This suggest that CR had no effect on injuries or mortality due to suspected or observed conflicts.

### 3.5. Reproductive Success

**Figure 4:**
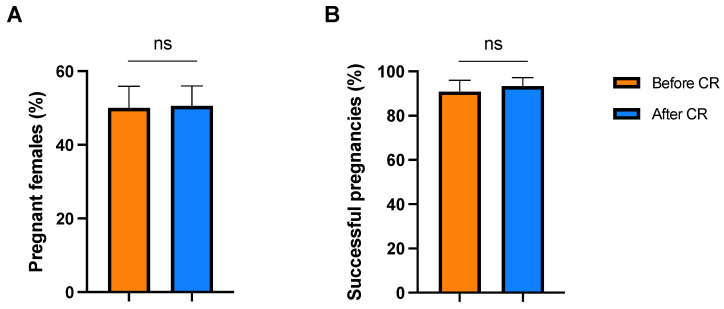
Proportion of pregnant females (**A**) and successful pregnancies (**B**) in a long-tailed macaque breeding colony before and after caloric restriction (CR). Error bars represent the standard error. ns: *p* ≥ 0.05.

Finally, we checked whether CR influenced female reproduction in the breeding colony. In the year prior to CR, 37 out of 74 (50%) fertile females conceived, while 44 out of 87 (51%) fertile females became pregnant in the year after CR (Figure 4A). The proportion of females that became pregnant did not significantly change after CR (chi-square test, χ^2^ = 0.005, *p* = 0.942). Female dominance had no effect on the likelihood to conceive both before CR (chi-square test, χ^2^ = 0.881, *p* = 0.644) and after CR (chi-square test, χ^2^ = 1.191, *p* = 0.551). Thirty out of 33 (91%) pregnancies resulted in viable offspring before CR, while 42 out of 45 (93%) pregnancies were successful after CR. The proportion of successful pregnancies did not significantly differ before or after CR (Fisher’s exact test, *p* = 0.694; Figure 4B). Thus, CR did not affect female reproduction.

## 4. Discussion

This study aimed to determine whether applying caloric restriction (CR) can be effective and safe in NHPs housed and fed in social groups. CR should ideally target overweight adults, while having no adverse effects on other group members. We investigated the effect of mild (20%) CR on adult overweight and biochemical parameters, immature growth, veterinary consultations, and reproductive success in multigenerational long-tailed macaque breeding groups. Overall, CR significantly reduced adult body measures and it had more effect on females and heavier individuals compared to males and leaner individuals. CR reduced cholesterol levels but did not affect overall triglyceride, glucose, fructosamine and HbA1c levels. Yet, biochemical parameters of individuals with high baseline values were reduced more compared to individuals with low baseline values. Immature growth, veterinary consultations and reproductive success remained equal after CR. Thus, mild CR can be considered effective and safe.

### 4.1. Adult Overweight and Biochemical Parameters

Overweight prevalence of full-grown adult male and female long-tailed macaques at BPRC combined was 22% before CR and 13% after CR. The WHI, used to evaluate overweight status, significantly decreased after CR. This complies with previous studies in individually- and pair-housed NHPs and indicates that CR is effective in reducing body weight. We also checked which adult individuals were affected most by CR. Delta WHI was independent of age and female dominance, but significantly related to baseline WHI and sex. Heavier individuals lost more weight compared to leaner individuals, which implies that CR targets the right individuals. Furthermore, WHI of females generally decreased, while four out of five males gained weight after CR. In contrast to our group-housed animals, who experience food competition, body weight reduction after CR was more pronounced in males compared to females in individually-housed rhesus macaques [38]. In the BPRC breeding colony, females likely experience more competition than males as groups contain multiple adult females and generally only one adult male, who is always top-ranking. This likely explains the sex-effect in our study. Alternatively, the small sample size for adult males (*n* = 5) may have caused this effect by chance.

CR reduced cholesterol levels of adults, but it had no overall effect on other biochemical parameters in our study. Yet, individuals with high baseline values had a higher reduction in their biochemical parameters after CR, i.e., were affected more by CR, compared to individuals with low baseline values. This again implies that CR targets the right individuals. This is partially consistent with findings from previous studies. Calorically restricted rhesus macaques had significantly lower cholesterol and triglyceride levels compared to ad libitum fed controls [39,40]. Biochemical parameters related to glycemic response, i.e., glucose, fructosamine and HbA1c, did not differ between CR- and control-fed long-tailed macaques [13]. Although a reduction in fasting glucose was not apparent immediately in rhesus macaques [41,42], glucose levels were significantly reduced after several years [43,44]. These previous studies generally evaluate the effect of 30–40% CR over several years, while we studied the effect of 20% CR after several months. This lower intensity and shorter duration may explain why we found no overall reduction in triglyceride and glucose levels after CR. Beneficial effects of CR on biochemical parameters may become more pronounced over time.

Thus, CR effectively reduced body weight measures and cholesterol levels of group-housed adult long-tailed macaques and had most effect on individuals with high baseline values for WHI and biochemical parameters.

### 4.2. Immature Growth

The suitability of CR in NHP breeding groups depends on whether CR is safe, which means there should be no adverse effects of CR on group members. Therefore, we first checked whether CR influenced immature body weight and height. Body weight and height increased with age but did not differ between the sexes. In free-ranging rhesus macaques, sex differences in body weight and height are first apparent in the age group 4–5 years old [37]. Sexual dimorphism occurs at a slightly younger age in rhesus and long-tailed macaques in captivity, i.e., between 3 and 4 years old [45]. This complies with our findings as our analyses included individuals between 8 months and 4 years old. Although sex itself had no effect, there was a significant interaction effect between age and sex in the models. The age-related increase in body weight and height differed per sex, and regression equations showed that males grew faster, both in weight and height, than females. Higher growth rates in males compared to females are also found in captive Japanese macaques, captive rhesus macaques and wild yellow baboons [21,46,47].

Despite the reduced amount of food, CR had no adverse effect on immature growth. Decreased growth rates could have occurred as immatures are generally less efficient in competitive foraging than adults [48]. However, many primates show high levels of feeding tolerance towards infants and juveniles [49,50]. Juveniles stealing food from other group members is even being tolerated in captive white-naped mangabeys (*Cercocebus lunulatus*: [51]). This high tolerance may enable immatures to obtain enough food and energy for growth, and likely explains why no effect of CR on immature growth was found.

### 4.3. Health Records and Reproductive Success

Health records were consulted in retrospect to obtain data on veterinary consultations and female reproduction. First, underweight and cases of severe weight loss were examined. Based on the ideal WHI range, underweight did not occur before CR, while one high-ranking female (2%) met the underweight criterion after CR. This was not considered a reason for concern, as this percentage fits within the normal variation [5]. Moreover, this female had similar reproductive output before and after CR and did not deviate in the number of births from other females. The proportion of individuals with severe weight loss did not differ before or after CR. Moreover, all cases were related to underlying medical conditions that are unlikely to be related to CR, i.e., diabetes or diarrhea. Actually, CR even prevents or delays the onset of type 2 diabetes mellitus and impaired glucose tolerance in captive male rhesus macaques [9]. In humans, diarrhea occurred equally often in participants on mild CR compared to participants on an ad libitum diet [52]. Thus, CR did not affect cases of severe weight loss as these were related to medical conditions that are likely not caused by CR.

Second, injuries and mortality due to suspected or observed conflicts were examined, as restricted resources may lead to increased levels of aggression. The relatively low number of injuries and mortality in this study imply that the breeding groups are socially stable in general. Although the cases of mortality were too limited for statistical testing, the proportion of individuals with injuries that required veterinary consultation did not differ before or after CR. This may be due to the provisioning of chow in multiple feeding trays per group, as this prevents monopolization and likely also aggression. Neither did our animal caretakers report higher levels of aggressive behaviour after CR (personal communication). These results suggest that CR had no effect on aggression. Similarly, aggressive behaviour did not change after a one-week 25% food reduction in a rhesus macaque group [53]. Behavioural observations on aggression were not performed in this study but are needed to confirm these results.

Third, the number of pregnancies and pregnancy outcomes were used to measure reproductive success. Females were equally likely to conceive and pregnancy outcomes did not differ before or after CR. Similar to our findings, female mouse lemurs housed in same-sex groups exposed to 40% CR for eight months became pregnant at similar rates and had an equal number of surviving offspring at weaning as control females [22]. Chronic CR also did not affect reproductive cycling and hormone concentrations in pair-housed female rhesus macaques [54]. Furthermore, the likelihood of conception was independent of female dominance before and after CR. In free-ranging Japanese macaques on Koshima island, birth rates were equal between females from different rank categories when artificial food supplies were abundant [55]. However, when artificial feeding was severely reduced, the most dominant females gave birth more frequently than the others [55]. Such a rank-effect was not found in our study, implying that CR equally influenced females from different rank categories. Altogether, this indicates that CR did not alter the reproductive success of the breeding colony.

### 4.4. Future Research

Although the number of calories fed were restricted by 20%, the fed amount was probably still enough to meet all individuals’ energetic requirements, i.e., malnutrition did not occur. Immature growth, veterinary consultations and female reproduction were unaffected by CR and females from different rank categories were equally likely to conceive. This indicates that we may have been overfeeding the colony before CR, while after CR caloric intake was closer to ‘optimal’. Similarly, a review on CR in NHPs concludes that the caloric intake of control animals is probably above ‘optimal’, i.e., they are generally overfed, compared to CR animals [56]. Using these animals in biomedical studies may bias experimental outcomes [57], so further investigation is needed to determine ‘optimal’ caloric intake for captive group-living NHPs.

This study provides the first evidence that mild CR may be a useful overweight management tool in captive group-living NHPs. However, several factors were not included in this study and should still be evaluated in group-housed NHPs, e.g., effects of CR on thermoregulation, stress response and behaviour. Some studies found physiological effects of CR. For example, core body temperature was ~0.5 °C lower in male rhesus macaques subjected to 30% CR compared to age-matched controls [58]. Besides, limited resources may elicit a stress response. In grey mouse lemurs, urinary cortisol concentrations increased after a two-week 60% CR [59]. Yet, more long-term studies found no evidence for a stress-related increase in cortisol levels in CR compared with control NHPs [60,61,62,63]. Whether CR triggers a stress response likely varies depending on its intensity and duration [59]. This and other effects of CR should be further studied in group-housed NHPs, as previous studies are limited to individually- and pair-housed NHPs.

## 5. Conclusions

Mild caloric restriction may be a valuable overweight management strategy in group-housed non-human primates as caloric restriction targeted the right individuals and had no adverse effects on the variables examined in this study.

## Figures and Tables

**Table 1:** The amount of food per individual in the diet of the long-tailed macaques at the Biomedical Primate Research Centre (BPRC) before and after mild caloric restriction (CR) was initiated in August 2017. The variation reflects variation in the amount of food for individuals of different ages and body weights.

Food Item	Before CR	After CR
Monkey chow	141 ± 36 g (range 79–294)	115 ± 37 g (range 65–265)
Fruit and vegetables	100/150 g	80/120 g
Bread	1 slice (~30 g)	1 slice (~30 g)
Grain mixture	20 g	15 g

**Table 2:** Mean ± SE (minimum–maximum) body measures and biochemical parameters of full-grown adult long-tailed macaques before and after mild caloric restriction (CR) started. * *p* < 0.05.

	*n*	Before CR	After CR
**Body measures**			
Body weight (kg)	46	6.7 ± 0.2 (4.25–11.5)	6.3 ± 0.2 (3.7–12.0) *
Abdominal circumference (cm)	38	43.2 ± 0.9 (29.4–56.9)	39.4 ± 0.9 (25.0–51.5) *
WHI (kg/m^2.7^)	46	57.2 ± 1.2 (40.5–78.4)	53.8 ± 1.0 (35.5–68.9) *
**Biochemical parameters**			
Cholesterol (mmol/L)	45	3.16 ± 0.11 (1.69–4.93)	2.96 ± 0.11 (1.53–4.74) *
Triglyceride (mmol/L)	45	1.46 ± 0.13 (0.35–4.67)	1.33 ± 0.14 (0.26–3.91)
Glucose (mmol/L)	45	3.56 ± 0.20 (1.67–7.61)	3.48 ± 0.24 (1.56–11.7)
Fructosamine (umol/L)	45	189 ± 3.5 (140–250)	187 ± 2.8 (153–244)
HbA1c (%)	12	4.37 ± 0.14 (3.81–5.59)	4.18 ± 0.07 (3.86–4.67)

## Data Availability

Data are available on request.

## Appendix B

**Table A1:** Health record examination of individuals with severe weight loss one year before and after caloric restriction (CR).

Case	Period	Age/Sex/Rank	Medical Condition
1	Before CR	12, M, high	Diabetes mellitus and diarrhea
2	Before CR	20, F, low	Diabetes mellitus
3	Before CR	12, F, high	Diarrhea
4	After CR	17, F, middle	Diabetes mellitus
5	After CR	13, F, high	Diarrhea
6	After CR	20, F, middle	Diabetes mellitus
7	After CR	22, F, low	Diarrhea
8	After CR	11, F, middle	Diarrhea
9	After CR	19, F, middle	Diabetes mellitus
10	After CR	24, F, low	Diarrhea
